# Prediction of Drug–Drug Interaction Potential of Tegoprazan Using Physiologically Based Pharmacokinetic Modeling and Simulation

**DOI:** 10.3390/pharmaceutics13091489

**Published:** 2021-09-16

**Authors:** Deok Yong Yoon, SeungHwan Lee, In-Jin Jang, Myeongjoong Kim, Heechan Lee, Seokuee Kim, Bongtae Kim, Geun Seog Song, Su-jin Rhee

**Affiliations:** 1 Department of Clinical Pharmacology and Therapeutics, College of Medicine and Hospital, Seoul National University, Seoul 03080, Korea; dyyoon18@snu.ac.kr (D.Y.Y.); leejh413@snu.ac.kr (S.L.); ijjang@snu.ac.kr (I.-J.J.); 2 Division of Clinical Development, HK inno.N Corporation, Seoul 04551, Korea; myeongjoong.kim@inno-n.com (M.K.); heechan.lee@inno-n.com (H.L.); seokuee.kim@inno-n.com (S.K.); bongtae.kim@inno-n.com (B.K.); geunseog.song@inno-n.com (G.S.S.); 3 Department of Pharmacy, College of Pharmacy, Wonkwang University, Iksan 54538, Korea

**Keywords:** tegoprazan, physiologically based pharmacokinetics, drug–drug interaction, CYP3A4, potassium-competitive acid blocker

## Abstract

This study aimed to develop a physiologically based pharmacokinetic (PBPK) model of tegoprazan and to predict the drug–drug interaction (DDI) potential between tegoprazan and cytochrome P450 (CYP) 3A4 perpetrators. The PBPK model of tegoprazan was developed using SimCYP Simulator^®^ and verified by comparing the model-predicted pharmacokinetics (PKs) of tegoprazan with the observed data from phase 1 clinical studies, including DDI studies. DDIs between tegoprazan and three CYP3A4 perpetrators were predicted by simulating the difference in tegoprazan exposure with and without perpetrators, after multiple dosing for a clinically used dose range. The final PBPK model adequately predicted the biphasic distribution profiles of tegoprazan and DDI between tegoprazan and clarithromycin. All ratios of the predicted-to-observed PK parameters were between 0.5 and 2.0. In DDI simulation, systemic exposure to tegoprazan was expected to increase about threefold when co-administered with the maximum recommended dose of clarithromycin or ketoconazole. Meanwhile, tegoprazan exposure was expected to decrease to ~30% when rifampicin was co-administered. Based on the simulation by the PBPK model, it is suggested that the DDI potential be considered when tegoprazan is used with CYP3A4 perpetrator, as the acid suppression effect of tegoprazan is known to be associated with systemic exposure.

## 1. Introduction

Tegoprazan is an acid suppression agent for the treatment of patients with acid-related diseases, including gastroesophageal reflux disease, peptic ulcer diseases, and *Helicobacter pylori* infection. The mechanism of acid suppression for tegoprazan is to reversibly inhibit gastric H^+^/K^+^-ATPase in a potassium-competitive manner [1]. In the phase 1 clinical study, tegoprazan up to 400 mg for a single dose and 200 mg for multiple doses was safe and tolerable for healthy adults, and the systemic exposure to tegoprazan increased in a dose proportional manner [2]. The mean half-life of tegoprazan is reported to be 3.7-6.2 h, and the apparent clearance and volume of distribution are reported to be approximately 17.6 L/h and 107.9 L, respectively [2,3,4]. The magnitude of acid suppression increases in a dose-dependent manner from 50 mg to 400 mg [2]. The approved dose of tegoprazan for acid-related diseases is 50 mg once daily.

The major metabolic pathway of tegoprazan is the liver, and a negligible amount is excreted by urine. Both in vitro and clinical results have elucidated that tegoprazan is a potential substrate of cytochrome P450 (CYP) 3A4. In the result of an in vitro study, ketoconazole, a strong inhibitor of CYP3A4, significantly inhibited the metabolism of tegoprazan in human liver microsomes, while other CYP inhibitors did not significantly affect the metabolic clearance of tegoprazan (in-house data). According to the label of tegoprazan, systemic exposure to tegoprazan increases when tegoprazan is co-administered with clarithromycin. Based on the in vitro and clinical data, it can be inferred that drug–drug interaction (DDI) between tegoprazan and CYP3A4 inhibitor may occur. However, the clinical DDI studies of tegoprazan conducted so far have been limited to the DDI between tegoprazan and clarithromycin or clarithromycin and amoxicillin, because tegoprazan is likely to be administered with these medications for *Helicobacter pylori* eradication [4]. Considering the substantial prevalence of acid-related diseases, tegoprazan is likely to be administered in combination with various drugs [5,6]. Therefore, further studies may be needed to assess the DDI between tegoprazan and other CYP3A4 perpetrators, which can affect both pharmacokinetics (PKs) and pharmacodynamics of tegoprazan by inducing or inhibiting the activity of CYP3A4. Nevertheless, it could be challenging to conduct clinical studies for all the cases of DDIs between tegoprazan and CYP3A4 perpetrators.

Physiologically based pharmacokinetic (PBPK) modeling is in silico mechanistic modeling combining the concept of the anatomical and physiological properties of the human body and the physicochemical and biological properties of a drug to simulate and predict the PK profile of the drug. Consequently, PBPK modeling and simulation can be applied to various steps in drug development [7]. The European Medicines Agency and the US Food and Drug Administration (FDA) published guidelines on PBPK modeling and simulation to manage PBPK qualification procedures intended for regulatory submission [8]. The simulation results from the PBPK model can contribute to regulatory decision making from a clinical pharmacology perspective, and the majority of applications of the PBPK approach in drug development focus on the prediction of the DDI [9,10,11]. Therefore, by constructing the PBPK model of tegoprazan, we can evaluate the DDI potential of tegoprazan as a substrate of CYP3A4. In other words, it is possible to quantitatively evaluate how the PKs of tegoprazan are altered.

Based on these understandings, the aim of the study was to develop and verify a PBPK model of tegoprazan and to apply the model to predict the DDIs between tegoprazan and CYP3A4 inhibitors or inducers by using commercially available PBPK modeling and simulation software.

## 2. Materials and Methods

### 2.1. Tegoprazan PBPK Model Development

**Figure 1:**
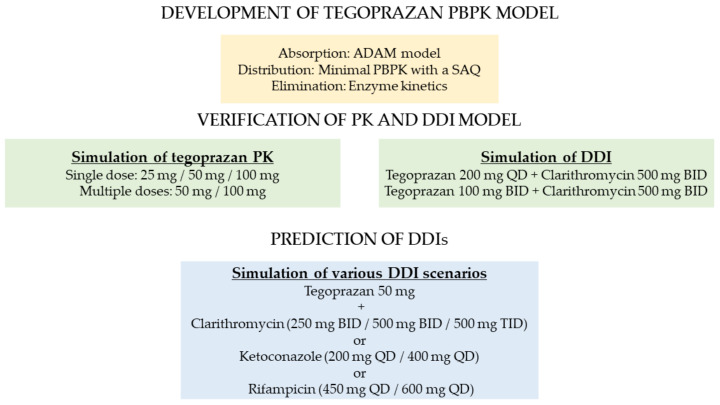
Overview of the tegoprazan physiologically based pharmacokinetic modeling process.

A PBPK model of tegoprazan was built and verified by both the bottom-up approach using in vitro data for maintaining a mechanistic PBPK structure and the top-down approach using clinical PK results for maintaining a descriptive structure (Figure 1). The initial PBPK model of tegoprazan was constructed using physicochemical properties (e.g., molecular weight, log P, pKa), in vitro data (e.g., permeability, intrinsic clearance), and in vivo data (e.g., renal clearance) provided by HK inno.N Corp. (Seoul, Korea). The commercially available software SimCYP simulator v19 (SimCYP Limited, Certara, Sheffield, UK) was used to build the PBPK model and to generate the PK simulations. The PBPK-model-predicted PK profiles and parameters of tegoprazan were compared with the observed PK profiles and parameters from previously conducted clinical studies [3,4,12] (Supplementary Table S1). The specific model configuration related to absorption, distribution, and elimination is described below.

#### 2.1.1. Absorption

The advanced dissolution, absorption, and metabolism model was used [13]. The unbound fraction of the drug in enterocytes (fu_Gut_) and the human jejunum effective permeability (P_eff,man_) were predicted because these values were not routinely measured (Table 1). The value of fu_Gut_ was predicted using the values of in vitro parameters, such as the octanol:water partition coefficient, the fraction of intracellular water, and other distribution-related parameters (in-house data). The value of P_eff,man_ was predicted using parallel artificial membrane permeation assay permeability (in-house data).

#### 2.1.2. Distribution

A minimal PBPK model with a single adjusted compartment (SAC) was used. The volume of distribution in the steady state was predicted using the method suggested by Rodgers and Rowland, based on the value of in vitro parameters (e.g., tissue neutral lipids, neutral phospholipids, tissue concentrations of acidic phospholipids, extracellular albumin) [14] (Table 1). The parameters for blood flow between the central compartment and SAC (Q) and the volume of SAC (V_SAC_) were included in the model to reflect the biphasic distribution of tegoprazan. The values of Q, V_SAC,_ and scalar applied to all predicted tissue Kp values (Kp scalar) were estimated to best describe the observed clinical data. 

#### 2.1.3. Elimination

The elimination of the PBPK model consisted of enzyme kinetic and renal clearance (Table 1). Intrinsic clearances (CL_int_) of tegoprazan by various CYPs were determined by an in vitro study that measured the fraction of CL_int_ inhibited by adding the inhibitors of CYP1A2, CYP2C9, CYP2C19, CYP2D6, and CYP3A to human liver microsomes (in-house data). Based on the in vitro data, an in vitro to in vivo extrapolation (IVIVE) approach was used to estimate the in vivo CL_int_ by each CYP enzyme [15]. Renal clearance as an additional clearance was used from the result of a single-oral-dose study of tegoprazan 100 mg.

### 2.2. Tegoprazan PBPK Model Refinement and Verification

The established PBPK model was verified by applying the predicted values to the clinical PK data from various phase 1 studies conducted with healthy male adults (Supplementary Table S2). Brief information about the clinical studies follows: study 1 (single-dose PK study), a single dose of tegoprazan 25 mg and 50 mg was orally administered; study 2 (food effect study), a single dose of tegoprazan 50 mg was orally administered in both fasted and fed states [12]; study 3 (bioequivalence study of two formulations), a single dose of two different formulations with tegoprazan 100 mg was orally administered [3]; study 4 (multiple-dose PK study), multiple doses of tegoprazan 50 mg and 100 mg were orally administered once daily for 7 days; study 5 (DDI study with clarithromycin), multiple doses of tegoprazan 200 mg were orally administered once daily with or without multiple doses of clarithromycin 500 mg twice daily for 5 days; and study 6 (DDI study with clarithromycin and amoxicillin), multiple doses of tegoprazan 100 mg were orally administered twice daily with or without multiple doses of clarithromycin/amoxicillin 500/1000 mg twice daily for 5 days or 7 days [4].

The PBPK model of tegoprazan as a single agent was verified using data from clinical studies of single- and multiple-dose administration of different dosages of tegoprazan. To verify the PK predictability of the PBPK model, the model-predicted PK profiles and parameters were compared with the observed PK profiles and parameters measured in clinical studies. The compared primary PK parameters were maximum plasma concentration (C_max_) and area under the plasma concentration–time curve (AUC) reflecting systemic exposure. When observed and predicted PK profiles were similar and the ratios of the predicted-to-observed PK parameters were between 0.5 and 2.0, we decided that the PBPK model was well constructed and the predictability of the PBPK was verified [16].

If the predicted PK profiles and parameters were not close enough to the observed values, the PBPK model was refined by the parameter estimation approach, in which a parameter was optimized with respect to the clinical data [17]. Parameter estimation was conducted using the genetic algorithm method and weighted-least squares as the objective function. Four parameters were simultaneously estimated in the final step of model refinement using clinical data of single-dose PK study of tegoprazan 50 mg (Table 1). The values of Q and V_SAC_ were estimated to reflect the biphasic distribution of tegoprazan, and the value of K_p_ scalar was estimated because it affected the overall PK profile, especially distribution and clearance. Furthermore, the value of in vivo CYP3A4 CL_int_ was also estimated instead of using in vitro data, to improve model fitting to the observed elimination profile, since the value of CYP3A4 CL_int_ was one of the most sensitive parameters affecting the PK profile of tegoprazan.

After refining and verifying the PBPK model of tegoprazan as a single agent, the DDI between tegoprazan and clarithromycin was finally verified using data from DDI clinical studies. To verify the predictability of the DDI estimated by the PBPK model of tegoprazan, the model-predicted PK profiles, parameters, and fold-increase of parameters were compared with the observed PK data measured in clinical studies (i.e., studies 5 and 6). In the case of study 6, the observed data were generated under the condition of triple administration of tegoprazan, clarithromycin, and amoxicillin. However, it was assumed that co-administration of amoxicillin does not affect the PKs of tegoprazan and clarithromycin because the DDI between tegoprazan and amoxicillin has been known to be negligible [4], and there was a low possibility of a DDI between amoxicillin and clarithromycin, considering the metabolic pathways of both drugs [18,19]. When simulating the DDI between tegoprazan and clarithromycin, the PBPK model of clarithromycin available in the SimCYP compound file was used.

All simulations for model verification were conducted using the same conditions as those used in the clinical studies, as follows: all subjects were healthy male volunteers aged 19–50 years, and tegoprazan and clarithromycin were both administered in fasted state. The output sampling interval in the SimCYP simulator tool box was set to 0.2 h in all simulations. Every clinical trial simulation was conducted in 10 trials with 10 subjects (total 100 subjects).

### 2.3. Prediction of a DDI Potential

A DDI potential between the approved dose of tegoprazan and three potent CYP3A4 perpetrators was simulated using the developed PBPK model of tegoprazan and PBPK models of clarithromycin, rifampicin, and ketoconazole available in the SimCYP compound files (Supplementary Table S2). The dosage regimens of tegoprazan, clarithromycin, ketoconazole, and rifampicin were selected based on the recommended daily doses on the drug labels. Clarithromycin and ketoconazole are well-known strong CYP3A4 inhibitors, and the maximum recommended daily doses are 500 mg three times a day and 400 mg a day, respectively [20,21]. Rifampicin is a well-known CYP3A4 inducer, and the maximum recommended daily dose is 600 mg a day [22].

The simulation was conducted using the same conditions as the conditions of model verification: all subjects were healthy male volunteers aged 19-50 years, and all drugs were assumed to be administered in fasted state. Tegoprazan PK profiles were predicted up to 192 h under the assumption that tegoprazan was administered alone or co-administered with perpetrators for 7 days. Every clinical trial simulation was conducted in 10 trials with 10 subjects (total 100 subjects). To evaluate the DDI potential of tegoprazan, the simulated PK profiles, PK parameters, and fold-increase PK parameters of tegoprazan with and without perpetrators were compared.

## 3. Results

### 3.1. PK Predictions of Tegoprazan

**Figure 2:**
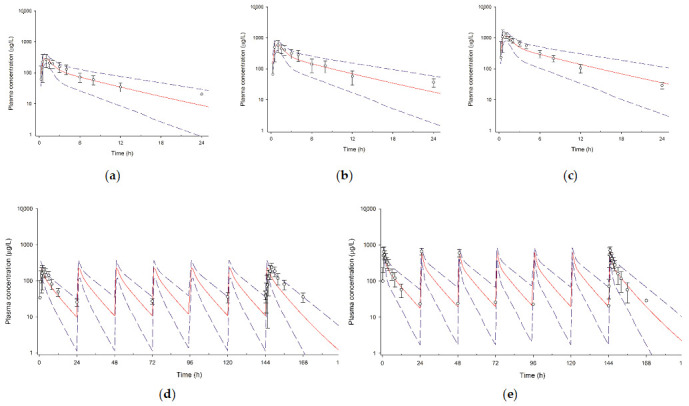
Observed and physiologically based pharmacokinetic-model-predicted plasma concentrations of tegoprazan in healthy subjects after single and multiple oral administration. The open circles and error bars represent the measured concentrations of tegoprazan and the standard deviations, respectively. The solid red lines and the dashed blue lines represent the simulated mean time-concentration profiles and the 5th–95th percentile of the total virtual population, respectively. (**a**) 25 mg single, (**b**) 50 mg single, (**c**) 100 mg single, (**d**) 50 mg multiple, and (**e**) 100 mg multiple.

The final PBPK model of tegoprazan adequately predicted the PK profiles of tegoprazan after single- and multiple-dose administration. The biphasic time-concentration profiles of tegoprazan after single- and multiple-dose administration of tegoprazan were well predicted by the final PBPK model (Figure 2). In addition, all ratios of the predicted-to-observed PK parameters, including C_max_ and AUC, were between 0.5 and 2.0, indicating that the model reproduced properly the observed PKs of tegoprazan (Table 2). The model-predicted median fraction of tegoprazan metabolized by hepatic CYP enzymes was calculated as 0.92, of which the portion of hepatic CYP3A4 accounted for 0.73.

### 3.2. Performance of the PBPK Model in Predicting DDI

**Figure 3:**
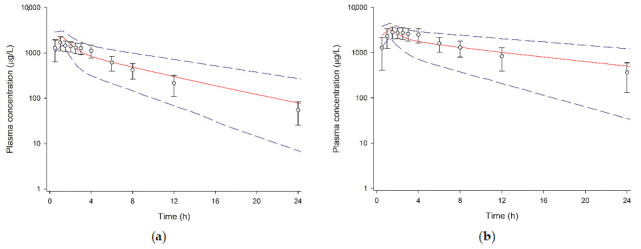
Observed and physiologically based pharmacokinetic-model-predicted plasma concentrations of tegoprazan following multiple oral administration of tegoprazan with and without clarithromycin. The open circles and error bars represent the measured concentrations of tegoprazan and the standard deviations, respectively. The solid red lines and the dashed blue lines represent the simulated mean time-concentration profiles and the 5th–95th percentile of the total virtual population, respectively. (**a**) Tegoprazan alone and (**b**) tegoprazan with clarithromycin.

The final PBPK model also predicted the DDI between tegoprazan and clarithromycin in that the model-predicted PK profiles of tegoprazan when tegoprazan was co-administered with clarithromycin were similar to the observed PK profile (Figure 3). The ratios of the predicted-to-observed PK parameters of tegoprazan were all between 0.5 and 2.0 when tegoprazan was administered with clarithromycin (Table 3). The model-predicted fold-increase of AUC during a dosage interval (AUC_τ_) for tegoprazan was similar to the observed value when tegoprazan was administered with clarithromycin; however, the fold-increase of C_max_ for tegoprazan was somewhat under-predicted (Table 3).

### 3.3. DDI Potential of Tegoprazan

**Figure 4:**
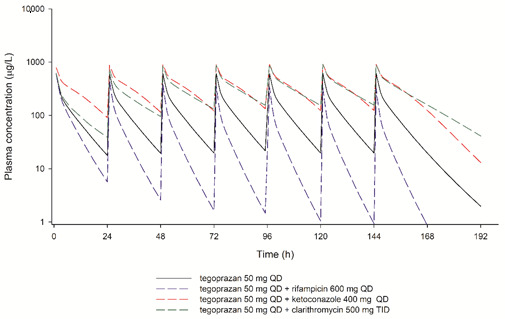
Physiologically based pharmacokinetic model-predicted plasma concentrations of tegoprazan when tegoprazan 50 mg was administered alone or with various CYP3A4 perpetrators for 7 days.

Systemic exposure to tegoprazan was expected to increase significantly when it was co-administered with the maximum recommended daily dose of clarithromycin or ketoconazole. In particular, the elimination profile of tegoprazan was continuously changed during multiple administrations with clarithromycin. However, when tegoprazan was co-administered with rifampicin, it was expected that tegoprazan elimination would gradually increase with multiple administrations, resulting in a decrease in systemic exposure (Figure 4). It was predicted that the AUC_τ,ss_ of tegoprazan will increase by approximately three times when tegoprazan 50 mg is administered with clarithromycin 500 mg three times a day or with ketoconazole 400 mg once a day for 7 days. Conversely, the AUC_τ,ss_ was predicted to decrease to approximately 30% when tegoprazan 50 mg was administered with rifampicin 600 mg once a day for 7 days (Table 4).

## 4. Discussion

In this study, we constructed the first PBPK model of tegoprazan for predicting DDIs by comprehensively applying physicochemical and PK properties of tegoprazan, including absorption, distribution, metabolism, and elimination data. Because tegoprazan shows dose proportional PKs, the PKs of tegoprazan could be predicted well in various dose strengths with single- and multiple-dose administration [2]. The tegoprazan PBPK model properly implemented the previously reported PKs of tegoprazan. The overall time-concentration profiles and PK parameter predictions were similar to clinical data in various dosing conditions (Figure 2 and Table 2). For example, the predicted exposure indices (i.e., C_max_ and AUC) for single or repeated administration of tegoprazan were consistent with the results reported in previous clinical studies, satisfying the 2-fold criteria that is commonly used in IVIVE prediction [16]. The predicted range of time to reach C_max_ was also comparable with the observed range in each trial [2,3,4,12,23]. In addition, the mean apparent clearance (i.e., AUC/dose) was predicted to be 17.5 L/h when tegoprazan was administered alone and decreased to 6.4 L/h by the co-administration of clarithromycin, which is similar to the results of the DDI study between tegoprazan and clarithromycin (17.7 L/h and 6.6 L/h, respectively) [4]. The clinical data used for model verification covered all dose ranges and regimens from previously reported clinical trials. Therefore, it was considered that the developed PBPK model is robust and can be used to predict the PKs of tegoprazan as well as DDI potentials by CYP3A4 perpetrators.

Tegoprazan is mainly metabolized by the liver, especially CYP3A4, and the administration of tegoprazan with clarithromycin triggers an increase in systemic exposure to tegoprazan because clarithromycin inhibits the activity of CYP3A4 [4]. The metabolic effects of other CYP enzymes, such as CYP1A2, CYP2C9, CYP2C19, and CYP2D6, on tegoprazan were not significant in in vitro studies (in-house data). Information about intrinsic clearance by CYP3A4 and other CYP enzymes was reflected in the final PBPK model, mechanistically enabling the prediction of DDIs. In DDI simulation results, the mean predicted total clearance was 16.0 L/h when tegoprazan was administered alone but decreased to 9.6 and 5.7 L/h by the combination of clarithromycin and ketoconazole, respectively. In addition, when tegoprazan was administered with rifampicin, the total clearance increased to 47.0 L/h. Along with these changes in total clearance by DDIs, the predicted hepatic CYP3A4 fraction metabolizing tegoprazan was changed from approximately 70% to 10% and 90% by the co-administration of CYP3A4 inhibitor (i.e., ketoconazole or clarithromycin) and inducer (i.e., rifampicin), respectively.

One advantage of PBPK modeling in predicting DDI is that the phenomenon of DDI can be interpreted mechanistically because the PBPK model is generally constructed based on various concepts of DDI, such as competitive inhibition and mechanism-based drug interaction. Especially, prediction of the effect of CYP3A4 perpetrators on the PKs of the substrate using the PBPK approach has been widely researched, and the PBPK-predicted and observed DDIs related to CYP3A4 metabolism were highly consistent [24,25]. Another advantage of PBPK modeling in predicting DDI is the ability to generate PK profiles for various dosages for which clinical DDI have not been tested. Although clinical DDI studies were performed only for tegoprazan 100 mg and 200 mg, the DDI could be predicted for the approved tegoprazan dose of 50 mg using the simulation based on the PBPK model in this study. It is known that the ability of a potassium-competitive acid blocker (P-CAB) such as tegoprazan to suppress acid is correlated to the PKs [2,26]. Therefore, by using the PBPK model of tegoprazan constructed in this study, DDIs between tegoprazan and CYP3A4 perpetrators can be predicted without conducting unnecessary clinical studies and the results of the prediction might be considered by clinicians to make decisions when prescribing tegoprazan with possible interacting drugs.

According to the guidelines for clinical drug interaction studies released by the FDA, a strong perpetrator refers to an inhibitor or an inducer that increases the AUC of a substrate by ≥5-fold or decreases the AUC of a substrate by ≥80%, respectively [27]. In this study, ketoconazole, clarithromycin, and rifampicin were selected as CYP3A4 perpetrators because these three drugs are well-known strong CYP3A4 perpetrators and widely applied to PBPK modeling and simulation for predicting DDI [20,21,22]. In the simulation for predicting DDI potential, the duration of administration of tegoprazan and CYP3A4 perpetrator was set to 7 days, since it is known that CYP3A4 enzymes can be induced or inhibited sufficiently by administering these drugs for 7 days [21,22]. Consequently, by simulating a scenario where tegoprazan was co-administered with CYP3A4 perpetrators in the maximum recommended daily dose, the changes in tegoprazan PK profiles in the worst-case scenario could be predicted. 

Based on the definition from the guideline, a moderately sensitive substrate is a drug whose AUC increases 2- to <5-fold when a strong index inhibitor is co-administered [27]. Accordingly, tegoprazan is considered a moderately sensitive substrate of CYP3A4 because the AUC of tegoprazan increases by up to about three times when ketoconazole or clarithromycin is co-administered. Moreover, the AUC of tegoprazan decreases to approximately 30% when rifampicin is co-administered. Therefore, if tegoprazan is administered with potential CYP3A4 perpetrators, a clinician might consider the potential DDI and refer to the simulation results.

The predicted ratio of increased AUC was similar to the observed values in both DDI studies, while the fold increase for C_max_ seems to have been under-predicted (Table 3). The under-estimated fold increase for C_max_ might be due to the variability in the data observed in clinical studies, considering that the values of C_max_ after multiple administration were lower than those after single administration. A possible reason for the decrease in C_max_ after multiple doses is pH-dependent change in the absorption of tegoprazan, that is, the C_max_ of tegoprazan might be reduced after multiple administrations due to augmented gastric pH caused by tegoprazan itself. In previous studies, when tegoprazan was administered with food, a decreased C_max_ was observed with a delayed time to reach C_max_, which was explained by an increase in gastric pH as food dilutes the H^+^ concentration in the stomach [12,28]. Because pH-dependent absorption was not reflected in the PBPK model, the difference between the observed and predicted C_max_ might have occurred. However, despite the under-predicted fold increase of C_max_, the magnitude of acid suppression can be inferred using AUC because the acid suppression ability of P-CAB is correlated to AUC rather than C_max_ [2,26].

When tegoprazan was administered with CYP3A4 perpetrators at the maximum recommended daily dose, the induction and inhibition profiles of CYP3A4 for tegoprazan were different based on the characteristics of the induction and inhibition mechanism (Figure 4). It takes time for endogenous enzymes to be fully induced because the transcription and translation of the enzyme are needed [29]. Therefore, systemic exposure to tegoprazan was gradually reduced when tegoprazan was administered with rifampicin. In the case of CYP enzyme inhibition, co-administration of tegoprazan and ketoconazole resulted in a rapid CYP3A4 inhibition profile, while co-administration with clarithromycin resulted in a gradual CYP3A4 inhibition profile. The phenomenon of gradual CYP3A4 inhibition profile might be caused by the fact that clarithromycin simultaneously acts as an inhibitor as well as a substrate of CYP3A4. Indeed, the mechanism-based inhibition of clarithromycin as a CYP3A4 perpetrator and substrate was reflected in the compound file of clarithromycin available in SimCYP and implemented in simulations for predicting DDIs between tegoprazan and clarithromycin [30].

One of the limitations in developing the PBPK model of tegoprazan in this study is that the predictability of DDIs of tegoprazan with ketoconazole and rifampicin was not verified since clinical DDI studies on tegoprazan and such drugs were not conducted. Nevertheless, since the predictability of the DDI between tegoprazan and clarithromycin was verified, it is considered that the model reflecting tegoprazan as a substrate of CYP3A4 would reasonably have predicted DDIs between tegoprazan and other CYP3A4 perpetrators. Another limitation of the PBPK model is that the properties of tegoprazan as a substrate of transporters or perpetrators of CYP enzymes were not reflected in the PBPK model. Some P-CABs, such as vonoprazan, potentially inhibit CYP2C19 at clinical doses [31], while the inhibition activity of tegoprazan against CYP2C19 was not evaluated through clinical study. If the additional data are generated through either in vitro or clinical studies and reflected in the model, the PBPK model of tegoprazan could be refined more sophisticatedly.

The PKs of tegoprazan has been investigated previously in various dosage ranges, and DDI with clarithromycin and food effect studies have also been performed [2,3,4,12,23]. However, there are still many aspects of PKs of tegoprazan unidentified mechanistically and clinically. It is impossible to conduct clinical trials for all scenarios to determine the PK properties of tegoprazan in an infinite number of clinical situations. In this regard, the tegoprazan PBPK model developed in this study helps to mechanistically simulate PK properties and DDI potentials for various dosing regimens with CYP enzyme perpetrators, without having to conduct clinical trials. In addition, the information simulated using the model can be used as evidence for appropriate drug therapy in clinical settings. This study focused on the DDI potential of CYP3A4 enzyme perpetrators, as tegoprazan is known to be primarily metabolized by CYP enzymes and is expected to be affected by CYP3A4 inhibitors. Although changes in the PKs of tegoprazan by clarithromycin have been reported in clinical trials [4], the doses used in the trials did not reflect the approved dose, and other situations, including the effect of CYP3A4 inducer on the PKs of tegoprazan, have not been identified. In this study, by developing a tegoprazan PBPK model, we have suggested that caution be used when using tegoprazan with potent CYP3A4 inhibitors or inducers. Our model also successfully predicted the metabolic profile of tegoprazan mechanistically, accounting for changes in the fraction metabolized by each CYP enzyme when tegoprazan was administered alone or in combination with CYP enzyme perpetrators. These results deepen the understanding of tegoprazan PKs, especially in terms of elimination aspects. The tegoprazan model presented in this study can be used as a basic model for the development of more sophisticated models to predict the pH-dependent absorption pattern of tegoprazan, food effects, or the effects of other perpetrators on metabolic enzymes and transporters.

## 5. Conclusions

In conclusion, the final PBPK model of tegoprazan as a substrate of CYP3A4 was successfully established and adequately predicted the DDI between tegoprazan and clarithromycin. Using this model, the PKs of tegoprazan can be mechanistically predicted, and the DDI potential under various clinical conditions can be predicted. Consequently, as a valid model, the PBPK model of tegoprazan developed through the study can be applied to the evidence-based dosing strategy by clinicians.

## Figures and Tables

**Table 1:** Parameter values used for the physiologically based pharmacokinetic model of tegoprazan.

Parameters and Models		Value	Source
Physiochemical properties	MW	387.38	Experimental data
	Log P	3	Experimental data
	pKa	Ampholyte	Experimental data
		pKa 1: 5.2	
		pKa 2: 12	
	B/P	0.868	Experimental data
	fu	0.124	Experimental data
Absorption	ADAM model	Data	Data
	fu_Gut_	0.008	Predicted using method 2 (Rodgers and Rowland 2007)
	P_eff,man_	12.397	Predicted using PAMPA permeability data
	PAMPA	68.4	Experimental data
Distribution	Minimal PBPK model + SAC		
	V_ss_	1.128	Predicted using method 2 (Rodgers and Rowland 2007)
	Q	24.4	Estimated
	V_SAC_	0.66	Estimated
	Kp scalar	0.33	Estimated
Elimination	CYP1A2 CL_int_	2.5	Experimental data
	CYP2C9 CL_int_	2.6	Experimental data
	CYP2C19 CL_int_	3.6	Experimental data
	CYP2D6 CL_int_	2	Experimental data
	CYP3A4 CL_int_	30.34	Estimated
	CL_R_	1.31	Experimental data

MW, molecular weight (g/mol); Log P, octanol:water partition coefficient; pKa, acid dissociation constant; B/P, blood:plasma partition ratio; ADAM, advanced dissolution absorption metabolism; fu, faction unbound in plasma; fu_Gut_, unbound fraction of drug in enterocytes; P_eff,man_, human jejunum effective permeability (10^−4^ cm/s); PAMPA, permeability measured by parallel artificial membrane permeability assay (10^−6^ cm/s); SAC, single adjusted compartment; V_SAC_, volume of the single adjusted compartment (L/kg); Q, blood flow (L/h); V_ss_, volume of distribution in the steady state (L/kg); Kp, scalar applied to all predicted tissue Kp values; CL_int_, intrinsic clearance (μL/min/mg of protein); CL_R_, renal clearance (L/h).

**Table 2:** A summary of observed and predicted pharmacokinetic parameters of tegoprazan using the final physiologically based pharmacokinetic model.

Treatment	Dose (mg)	*n*		T_max_ (h) *		C_max_ (μg/L)			AUC_inf_ or AUC_τ_ (μg∙h/L) **		
		Pred.	Obs.	Pred.	Obs.	Pred.	Obs.	Ratio (Pred./Obs.)	Pred.	Obs.	Ratio (Pred./Obs.)
Single oral dose	25	100	12	0.95	0.75	310.4	335.6	0.92	1479.4	1340.0	1.03
				(0.50–1.62)	(0.50–3.00)						
	50	100	24	0.95	1.00	620.6	759.1	0.82	2958.6	2903.0	1.02
				(0.50–1.62)	(0.50–2.00)						
	100	100	12	0.95	1.00	1241.2	1434.5	0.87	5916.6	5998.1	0.99
				(0.50–1.62)	(0.50–1.00)						
Multiple oral doses ^†^	50	100	6	0.94	1.00	638.9	842.8	0.76	2969.5	2954.9	1.00
				(0.51–1.59)	(0.50–1.03)						
	100	100	6	0.95	1.25	1277.6	1149.7	1.11	5929.4	4768.4	1.24
				(0.50–1.58)	(0.50–3.00)						

T_max_, the time to reach the maximum plasma concentration; C_max_, maximum plasma concentration; AUC_inf_, area under the concentration–time curve from time zero to infinity; AUC_τ_, area under the concentration–time curve from time zero to 24 h concentration; Pred., predicted data; Obs., observed data. Data are presented as the mean. * T_max_ is expressed as the median (range). ** AUC_inf_ and AUC_τ_ were evaluated followed by single and multiple administration, respectively. ^†^ Multiple oral doses of tegoprazan were administered once daily for 7 days.

**Table 3:** Fold increase of systemic exposure to tegoprazan when co-administered with clarithromycin or clarithromycin/amoxicillin.

Treatment	*n*		T_max_ (h) *		C_max_ (μg/L)			AUC_τ_ (μg∙h/L)			Fold Increase			
	Pred.	Obs.	Pred.	Obs.	Pred.	Obs.	Ratio (Pred./Obs.)	Pred.	Obs.	Ratio (Pred./Obs.)	Pred. C_maxR_	Obs. C_maxR_	Pred. AUC_R_	Obs. AUC_R_
T 200 mg QD ^†^	100	24	0.95	1.00	2554.8	1868.6	1.37	11,838.9	10,817.6	1.09				
			(0.50–1.58)	(0.50–4.00)										
T 200 mg QD + C 500 mg BID ^†^	100	24	1.04	1.50	3491.4	3096.0	1.13	28,881.4	27,796.4	1.04	1.37	1.66	2.44	2.57
			(0.55–1.62)	(1.00–4.00)										
T 100 mg BID ^††^	100	20	0.95	1.30	1411.3	1018.4	1.39	5921.6	5955.9	0.99				
			(0.51–1.55)	(0.50–6.00)										
T 100 mg BID + C 500 mg BID + A 1000 mg BID ^†††^	100	20	1.03	2.50	2268.2	2285.6	0.99	14,897.5	16,045.0	0.93	1.61	2.24	2.52	2.69
			(0.55–1.55)	(1.00–3.00)										

T, tegoprazan; C, clarithromycin; A, amoxicillin; QD, once daily; BID, twice daily; T_max_, the time to reach the maximum plasma concentration; C_max_, maximum plasma concentration; AUC_τ_, area under the concentration–time curve from time zero to 24 h concentration; Pred., predicted data; Obs., observed data; C_maxR_, ratio of increased maximum plasma concentration; AUC_R_, ratio of increased area under the concentration–time curve from time zero to 24 h. Data are presented as the mean. * T_max_ is expressed as the median (range). ^†^ Tegoprazan 200 mg once daily without or with clarithromycin 500 twice daily was administered for 5 days. ^††^ Tegoprazan 100 mg twice daily for 4 days and tegoprazan 100 mg once daily on day 5 were administered. ^†††^ Tegoprazan 100 mg twice daily with amoxicillin 1000 mg/clarithromycin 500 mg twice daily for 6 days and tegoprazan 100 mg once daily with amoxicillin 1000 mg/clarithromycin 500 mg once daily on day 7 were administered.

**Table 4:** Prediction of systemic exposure changes of tegoprazan 50 mg with co-administration of the perpetrator using the final physiologically based pharmacokinetic model.

Perpetrator	Predicted C_max_ (μg/L)	Predicted AUC_τ_ (μg∙h/L)	Predicted Fold Increase	
			C_maxR_	AUC_R_
Clarithromycin 250 mg BID	768.7	4896.3	1.20	1.63
Clarithromycin 500 mg BID	887.8	7455.8	1.40	2.57
Clarithromycin 500 mg TID	933.5	8356.4	1.47	2.96
Ketoconazole 200 mg QD	905.8	7633.2	1.44	2.84
Ketoconazole 400 mg QD	936.2	8382.8	1.49	3.14
Rifampicin 450 mg QD	367.8	931.7	0.57	0.31
Rifampicin 600 mg QD	353.7	873.5	0.55	0.29

QD, once daily; BID, twice daily; TID, three times a day; C_max_, maximum plasma concentration; AUC_τ_, area under the concentration–time curve from time zero to 24 h; C_maxR_, ratio of increased maximum plasma concentration; AUC_R_, ratio of increased area under the concentration–time curve from time zero to 24 h.

## Data Availability

The data sets generated and/or analyzed during the current study are available from the corresponding author on reasonable request.

## Supplementary Materials

The following are available online at https://www.mdpi.com/article/10.3390/pharmaceutics13091489/s1, Electronic Supplementary Materials: Additional information on clinical studies and simulation outline including Tables S1 and S2.
