# Supplementary material for: Prediction of Drug–Drug Interaction Potential of Tegoprazan Using Physiologically Based Pharmacokinetic Modeling and Simulation
Source: Pharmaceutics. 2021 Sep 16;13(9):1489. doi: 10.3390/pharmaceutics13091489 (PMC8464955; doi:10.3390/pharmaceutics13091489)
Supplement: Supplementary file 1 [file pharmaceutics-13-01489-s001.zip › pharmaceutics-1361126-supplementary.pdf]

pharmaceutics

Table S1. Summary of information on clinical studies of tegoprazan.

Study No.	Study design	No. of subjects	Dose regimen of tegoprazan	PK sampling time	Reference*
Study 1	Single-dose pharmacokinetic study	12	25 mg / 50 mg single	up to 48 h	NCT03530228
Study 2	Food effect study	12	50 mg single	up to 48 h	NCT03863938
Study 3	Bioequivalence study of two formulations	12	100 mg single	up to 48 h	NCT02995239
Study 4	Multiple-dose pharmacokinetic study	6	50 mg /100 mg QD for 7days	up to 48 h	NCT03009760
Study 5	DDI study with clarithromycin	24	200 mg QD for 5 days	up to 48 h	NCT02052336
Study 6	DDI study with clarithromycin and amoxicillin	24	100 mg BID for 5 days or 7 days	up to 48 h	NCT03011996

QD, once daily; BID, twice daily. *References are ClinicalTrials.gov Identifier.

Table S2. Simulation outline of tegoprazan single- and multiple-dose pharmacokinetic and drug-drug interaction studies.

Tegoprazan	Dose (mg)	Treatment Day	Interacting Drug	Dose (mg)	Treatment Day	Analysis
Single dose	25	1	-	-	-	Pred. versus Obs.
	50	1	-	-	-	Pred. versus Obs.
	100	1	-	-	-	Pred. versus Obs.
Multiple dose	50 QD	7	-	-	-	Pred. versus Obs.
	100 QD	7	-	-	-	Pred. versus Obs.
Multiple dose with interacting drug	200 QD	5	Clarithromycin	500 BID	5	Pred. versus Obs.
	100 BID	5/7*	Clarithromycin	500 BID	7	Pred. versus Obs.
	50 QD	7	Clarithromycin	250 BID	7	Pred.
	50 QD	7	Clarithromycin	500 BID	7	Pred.
	50 QD	7	Clarithromycin	500 TID	7	Pred.
	50 QD	7	Ketoconazole	200 QD	7	Pred.
	50 QD	7	Ketoconazole	400 QD	7	Pred.
	50 QD	7	Rifampicin	450 QD	7	Pred.
	50 QD	7	Rifampicin	600 QD	7	Pred.

QD, once daily; BID, twice daily; TID, three times a day, Pred., Predicted data; Obs., Observed data. *When tegoprazan was administered alone, tegoprazan was administered for 5 days, while, when tegoprazan was co-administered with clarithromycin, tegoprazan was administered for 7 days.
